# Hate speech detection in the Arabic language: corpus design, construction, and evaluation

**DOI:** 10.3389/frai.2024.1345445

**Published:** 2024-02-20

**Authors:** Ashraf Ahmad, Mohammad Azzeh, Eman Alnagi, Qasem Abu Al-Haija, Dana Halabi, Abdullah Aref, Yousef AbuHour

**Affiliations:** ^1^Department of Computer Science, Princess Sumaya University for Technology (PSUT), Amman, Jordan; ^2^Department of Data Science, Princess Sumaya University for Technology (PSUT), Amman, Jordan; ^3^Department of Cybersecurity, Faculty of Computer and Information Technology, Jordan University of Science and Technology, Irbid, Jordan; ^4^SAE Institute, Luminus Technical University College (LTUC), Amman, Jordan; ^5^Department of Basic Sciences, Princess Sumaya University for Technology (PSUT), Amman, Jordan

**Keywords:** Arabic hate speech, natural language processing (NLP), machine learning, Arabic hate speech detection, Arabic hate speech corpus

## Abstract

Hate Speech Detection in Arabic presents a multifaceted challenge due to the broad and diverse linguistic terrain. With its multiple dialects and rich cultural subtleties, Arabic requires particular measures to address hate speech online successfully. To address this issue, academics and developers have used natural language processing (NLP) methods and machine learning algorithms adapted to the complexities of Arabic text. However, many proposed methods were hampered by a lack of a comprehensive dataset/corpus of Arabic hate speech. In this research, we propose a novel multi-class public Arabic dataset comprised of 403,688 annotated tweets categorized as extremely positive, positive, neutral, or negative based on the presence of hate speech. Using our developed dataset, we additionally characterize the performance of multiple machine learning models for Hate speech identification in Arabic Jordanian dialect tweets. Specifically, the Word2Vec, TF-IDF, and AraBert text representation models have been applied to produce word vectors. With the help of these models, we can provide classification models with vectors representing text. After that, seven machine learning classifiers have been evaluated: Support Vector Machine (SVM), Logistic Regression (LR), Naive Bays (NB), Random Forest (RF), AdaBoost (Ada), XGBoost (XGB), and CatBoost (CatB). In light of this, the experimental evaluation revealed that, in this challenging and unstructured setting, our gathered and annotated datasets were rather efficient and generated encouraging assessment outcomes. This will enable academics to delve further into this crucial field of study.

## 1 Introduction

**Figure 1:**
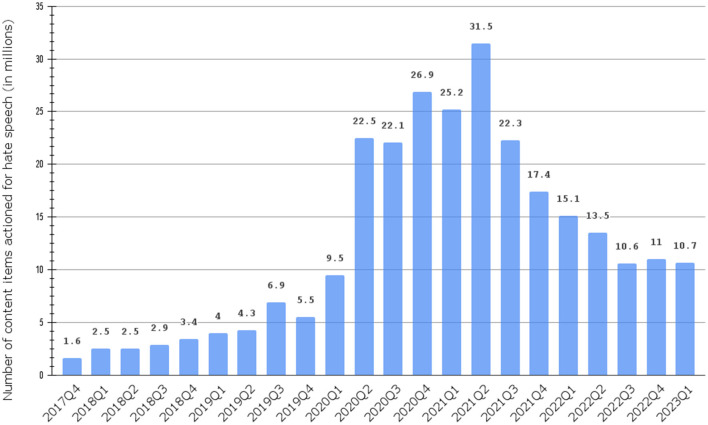
Number of content items actioned for hate speech on Facebook worldwide between 4th quarter 2017 and 1st quarter 2023.

In recent years, the spread, diversity, and ease of use of social media platforms (e.g., Facebook, Twitter, etc.) have facilitated the rapid dissemination of information and the quick growth of virtual communities (Kapoor et al., 2018). Social media has changed the typical daily Routines of individual traditional business operations and interaction patterns within various communities (Ngai et al., 2015). Despite the benefits of these advances, individuals and communities became vulnerable to new forms of harm and verbal aggression that were not common before. Hate speech has gained prominence as a form of discourse that targets individuals or groups based on race, religion, gender, sexual orientation, or other characteristics (Yalçınkaya, 2022). The number of content items on which Facebook took action due to hate speech worldwide between the 4th quarter of 2017 and the 1st quarter of 2023 is presented in Figure 1. Despite the decrease in numbers as governments worldwide relaxed COVID-19-related constraints, the number in the first quarter of 2023 is higher than the corresponding interval of 2020 and more than double the number of the corresponding interval of 2019.^1^

Different forms of hate speech include harassment, cyberbullying, offense, and abuse (Fortuna et al., 2020; Omar et al., 2020). Harassment refers to persistent unwanted behavior that causes distress or fear, often involving repetitive and intrusive actions (Gilani et al., 2014). Cyberbullying specifically occurs in the digital realm, encompassing the use of technology to harass, intimidate, or demean others (Coban et al., 2023). Offense refers to actions or expressions that cause displeasure or upset, while abuse involves using power or authority to harm or control others (Husain, 2020).

Many recent studies have shown the relationship of hate speech to the increase in hate crimes worldwide (Nguyen, 2023). It also showed its connection to the exposure of targeted individuals to discrimination, violations, and denial of their human rights (Chakraborty and Masud, 2022).

Social media can be very helpful for connecting people, increasing self-esteem (Zsila and Reyes, 2023), and being a platform for information exchange and self-expression (Siddiqui et al., 2016). Other use of social media in societies includes, but are not limited to, building communities and helping in emergencies (Akram and Kumar, 2017). On the other hand, social media may hurt mental health as it may lead to stress, increased sadness and isolation (Zsila and Reyes, 2023), and addiction, as well as the possibility of hurting privacy and security, facilitating fraud (Akram and Kumar, 2017), the spread of misinformation and hate speech. Social media has been used successfully in business for marketing, identifying, and engaging talents (Akram and Kumar, 2017). Other use of social media in business includes, but are not limited to, customer support, facilitating communications between employees, and employee self-development (Siddiqui et al., 2016). Furthermore, social media has been found to have a positive value in higher education, particularly as a teaching and learning tool (Sobaih et al., 2016); it can increase peers' interactivity and online knowledge-sharing behavior which has a positive impact on students' engagement, can lead better academic performance (Ansari and Khan, 2020). Also, the use of social media was found to increase e-learning platform acceptance by students (Alghizzawi et al., 2019).

The propagation of hate speech online continuously challenges policy-makers and the research community due to difficulties limiting the evolving cyberspace, the need to empower individuals to express their opinions, and the delay of manual checking (Jahan and Oussalah, 2023).

To reduce its risks and possible devastating effects on the lives of individuals, families, and communities, the NLP community has shown an increasing interest in developing tools that help in the automatic detection of hate speech on social media platforms (Husain and Uzuner, 2021) as the detection of hate speech can be, generally, modeled as a supervised learning problem (Schmidt and Wiegand, 2017). Several studies investigated the problem and contrasted various processing pipelines using various sets of features and classification algorithms [e.g., Naive Bayes, Support Vector Machine (SVM), deep learning architectures, and so on] (Jahan and Oussalah, 2023).

Fairly generic features, such as a bag of words or embeddings, resulted in reasonable classification performance, and character-level schemes outperformed token-level approaches (Schmidt and Wiegand, 2017). It is reported in the literature that even though information derived from text can be useful for detecting hate speech, it may be beneficial to use some meta-information or information from other media types (e.g., images attached to messages) (Jahan and Oussalah, 2023).

While several studies investigated anti-social behaviors such as abusive or offensive language cyberbullying and heat speech, a limited number of researches have contributed to hate speech detection in Arabic in general (Al-Hassan and Al-Dossari, 2019). At the time of writing, we are unaware of any study attempting to detect hate speech for the Arabic dialect used in Jordan. Compared to English, Arabic could be more resourceful regarding NLP. The existence of different dialects combined with the richness and complexity of Arabic morphology add up more challenges to Arabic NLP research (Albadi et al., 2018).

The main contributions of this work are as follows:

1. Construct a public Arabic-Jordanian dataset of 403,688 annotated tweets labeled according to the appearance of hate speech as very positive, positive, neutral, and negative.
2. Comparing the performances of machine learning models for Hate speech detection of Arabic Jordanian dialect tweets.

The remainder of this paper is organized as follows. The related work to Jordanian dialect datasets and Arabic Hate speech detection are reviewed in Section 2. Section 3 details our methodology for constructing the new dataset, the preprocessing steps, and statistics. Section 4 describes in detail the architecture of classification models, the conducted experiments, and the results. Section 4 discusses and analyzes the results. Finally, Section 5 concludes our work and discusses future directions.

## 2 Literature review

With the rapid spread of social media platforms, the freedom level has been elevated so that many people can give their opinions with advice or criticism without borders. People with shy and conservative personalities have been allowed to speak up and give their opinions without fear of interruption or hesitation. The problem is that many people have abused this freedom by not considering the courtesy of speech and descent manners. Hate speech, including cyberbullying, offensive talk, sarcasm, and harassment, are just a few examples of freedom abuse on social media (Omar et al., 2020).

This problem has motivated researchers to create methods to detect and stop such violations that have a large negative influence on our societies, youth, and children. In this section, selective literature is introduced and discussed to illustrate the methods conducted in this area.

Surely, the problem of hate speech has been considered in several scopes: science, sociology, psychology, and even criminology. This research will concentrate on the technical efforts conducted in this area, i.e., Natural Language Processing (NLP) and Artificial Intelligence (AI), to detect such behavior.

### 2.1 Hate speech and related concepts

NLP is one of the common disciplines that is needed in the area of hate speech detection. Posts, tweets, comments, reviews, and most social contributions on social media are inserted as text. People from all over the world can express their feelings with their language and even dialect. No language standards are enforced on such platforms, and thus, NLP tools have become essential in representing, understanding, and analyzing these inputs.

AI algorithms, either Machine Learning (ML) or Deep Learning (DL) algorithms, have been extensively conducted as classification algorithms to detect hate speech in text extracted from social media (Husain and Uzuner, 2021; Yi and Zubiaga, 2023).

The most vital issue when tackling this problem is to work on a high-quality hate speech corpora. In literature, two streams are taken into consideration. Many researchers use public corporations directed to hate speech in general or in a certain type of hate. Such corpora can be hard to find, especially in low-resources languages. Thus, most of the literature that adopts this stream works on English corpora as in Mozafari et al. (2020), Aldjanabi et al. (2021), and Awal et al. (2021). Public Arabic hate speech corpora can also be found but rarely concentrate on certain Arabic dialects. Abuzayed and Elsayed (2020), Haddad et al. (2020), and Hassan et al. (2021), for example, have used in their research the OffensEval 2020 dataset, which shared task competition organizers have provided. In another case, Alsafari et al. (2020b) have proposed an Arabic hate speech corpus that they reused in further experiments in Alsafari et al. (2020a). Sections 2.2, 2.3 highlight literature that created hate speech corpora in different languages.

The next issue in this problem is representing the text (posts, tweets, comments, etc.) in proper text presentation (word embedding technique), enabling AI classifiers to handle them as proper inputs and thus produce the desired outputs. Any NLP task needs such text presentation methods. In literature, several word embedding techniques are used and, in some cases, compared in the same paper. Examples of such techniques are TF-IDF (Abuzayed and Elsayed, 2020), word2vec and some of its variations such as AraVec (Aref et al., 2020; Faris et al., 2020; Romim et al., 2021), and Fasttext (Alsafari et al., 2020a,b; Aref et al., 2020; Romim et al., 2021).

Using these corpora to detect hate speech on social media platforms is a classification problem that needs labeled data. Labeling of each text sample should be applied using either manual or automatic annotation processes. Number of classes varies from one research to another. Many papers use the binary classes by only labeling the samples with two labels. Hate or Not hate is the most common binary label used in literature, such as Alshaalan and Al-Khalifa (2020), Aref et al. (2020), Omar et al. (2020), Romim et al. (2021), Saeed et al. (2022), and Khezzar et al. (2023). Others used different labels for binary classification, such as clean or offensive (Alsafari et al., 2020b; Alsafari and Sadaoui, 2021), hateful or normal (Salomon et al., 2022). Some researchers were more precise in identifying the labels according to the type of hate speech detected. For example, in Mursi et al. (2022), Islamic Radicalism is the type of hate to detect, and thus the binary labels are extremist or non-extremist. In Coban et al. (2023), the target was to detect whether cyberbullying terms exist in Facebook posts; thus, the binary labels used were cyberbullying or non-cyberbullying.

Three-labeled corpora have been introduced, with a third label that either indicates a neutral label (Faris et al., 2020) or undecided (Ameur and Aliane, 2021). Also, the three labels have been used to distinguish between hate and abusive classes in addition to clean or normal, as in Alsafari et al. (2020b), Alshalan and Al-Khalifa (2020), and Duwairi et al. (2021).

Other research used multi-labeled corpora, including fine-grained labels that identify the type of hate detected more specifically. In Anezi (2022), the arHate Dateset has been created, with labels: racism, against religion, gender inequality, violence, offensive, and bullying. These labels have been selected to distinguish the precise type of hate speech. Additional labels were added to indicate the existence of hate speech other than the ones mentioned previously, using labels normal positive and normal negative. Other examples are Alsafari et al. (2020b), Ahmed et al. (2022), Beyhan et al. (2022), and Mollas et al. (2022), which used multi-labels to annotate their corpora, that in some cases reached eight different labels, according to how many details desired to be expressed in the labels.

Several classifiers have been used in the literature to apply the classification task. Most research compared different models to find the most proper one(s) for the created or the public corpora tested.

The classifiers used were categorized into ML and DL algorithms. In Abuzayed and Elsayed (2020), both ML and DL classifiers have been applied and compared. Fifteen traditional ML classifiers, such as SVM, RF, XGBoost, DT, LR, etc., have been used. On the other hand, DL classifiers have been used, such as CNN and RNN. When compared, it has been found that the best classifier was the hybrid CNN and RNN classifier.

In Althobaiti (2022), SVM and LR have been used and compared with a BERT-based model where the BERT model yielded the best results. In their research, a novel approach has been conducted by including emojis found in the tweets in the hate speech detection.

Mozafari et al. (2020) used the BERT model to propose their model. They added extra layers on BERT, consisting of CNN and LSTM.

The ensemble concept has been addressed in research since, in Alsafari et al. (2020a), the authors have created an ensemble model consisting of CNN and BiLSTM classifiers. They have compared this ensemble model with other individual models, and it outperformed the others.

In Sections 2.2, 2.3, research that proposed hate speech corpora is discussed and illustrated.

### 2.2 Arabic hate speech corpora and detection systems

Arabic, as a low-resource language, needs more specialized hate speech corpora. As aforementioned, research has been found and discussed in the previous sub-section, highlighting some research in this area. Nevertheless, Arabic dialects' hate speech datasets are not easily found in the literature.

This section discusses a sample of research that created Arabic hate speech corpora. Table 1 summarizes the main aspects of this sample.

**Table 1:** Summary of literature on Arabic hate speech corpora.

**References**	**Dialect**	**Source**	**Dataset size**	**Labeling process**	**Classes**	**Best classifier**	**Results**
Omar et al. (2020)	Mixed	Facebook, Twitter, Instagram, YouTube	20,000	Manual	Hate, Not hate	RNN	Acc: 98.7%, F1-score: 98.7%, Recall: 98.7%, Precision: 98.7%
Alshalan and Al-Khalifa (2020)	Saudi	Twitter	9,316	Manual	hateful, abusive, or normal	CNN	Acc: 83%, F1-score: 79%, Recall: 78%, Precision: 81%, AUC: 79%
Ameur and Aliane (2021) (AraCOVID19-MFH)	Mixed	Twitter	10,828	Manual	Yes, No, Cannot decide	Arabert Cov19	F1-score: 98,58%
Duwairi et al. (2021) (ArHS)	Levantine	Twitter	9,833	Manual	Hate or normal; hate, abusive or normal	Binary class: CNN, Ternary class: BiLSTM-CNN and CNN, Multi-class: CNN-LSTM and the BiLSTM-CNN	F1-score: 81%, F1-scode: 74%, F1-score: 56%
Faris et al. (2020)	Mixed	Twitter	3,696		Hate, neutral, or normal	LSTM+ CNN, with word embedding Aravec (N-grams and skip grams)	F1-score: 71.68%
Anezi (2022) (arHateDataset)	mixed	Variaty	4,203		Racism, Against religion, gender inequality, violence, offense, bullying, normal positive, and normal negative	RNN architectures: DRNN-1: binary classification, DRNN-2: multi-labeled classification	Validation accuracy: 83.22%, 90.30%
Khezzar et al. (2023)	Mixed	Twitter (public datasets)	34,107	Unifying annotation in datasets	Hate, no hate	AraBERT	Accuracy: 93%
Alsafari and Sadaoui (2021)	Standard and Gulf	Twitter	Training: 9,345, Unlabeled: 5M, Testing: 4,002	Semi-supervised learning	Clean, or offensive	CNN + Skip gram	F1-score: 88.59%, Recall: 89.60%, Precision: 87.69%
Aref et al. (2020)	Mixed	Twitter	3,232	Manual	Hate or Not hate	CNN-FastText	Acc: 71%, F1-score: 52%, Recall: 69%, Precision: 42%
Alshaalan and Al-Khalifa (2020)	Saudi	Twitter	9,316		Hate or not hate	CNN	Acc: 83%, F1-score: 79%, Recall: 78%, Precision: 81%
Salomon et al. (2022)	Tunisian	Twitter	10,000	Manual	Hateful or Normal	AraBERT	F1-Score: 99%
Alsafari et al. (2020b)	Gulf	Twitter	5,361	Manual	2-classes: Clean or Offensive/Hate, 3-classes: Clean, Offensive or Hate, 6-classes: Clean, Offensive, Religious Hate, Gender Hate, Nationality Hate or Ethnicity Hate	CNN + mBERT	2-classes: 87.03 %, 3-classes: 78.99%, 6-classes: 75.51%
Mursi et al. (2022)	Mixed	Twitter	3,000	Manual	Extremist or non-extremist	SVM	Acc: 92%, F1-score: 92%, Recall: 95%, Precision: 89%

Social media platforms have been considered the sanctuary of different types of people in society to express their feelings. Many people post their social news and events, either happy or sad, to the public. Nevertheless, this publicity can encourage some indecent people to reflect their negative feelings of hate, sarcasm, bullying, and others. Thus, social media platforms are considered the main resources of datasets, corpora, that consist of samples that can be trained and tested for the hate speech detection task.

In the literature, it has been found that Facebook (Omar et al., 2020; Ahmed et al., 2022; Anezi, 2022), Twitter, Instagram, and YouTube (Omar et al., 2020) are some of the main sources of such data. As illustrated in Table 1, most of the research used Twitter as the social media source; this indicates that this platform provides the data more easily to researchers than other platforms, such as Facebook. Another reason researchers prefer to collect data from Twitter is that tweets mostly consist of short text. While other platforms, such as Instagram or YouTube, consist of data in the form of images and videos, which takes more work to process. Also, some platforms, such as Facebook, Telegram, and Reddit, may have text content, but in most cases, the text is long and can take longer to process.

The Arabic language has many challenges when processed and tackled. Yet, standard Arabic has its rules and grammar that can make the text understanding and analysis easier. Arabic dialects, on the other hand, propose a hard problem for AI to distinguish and understand. Thus, collecting Arabic dialect data has been a hot research topic that Arabian authors have considered when conducting NLP tasks, specifically hate speech detection.

As illustrated in Table 1, many researchers collected data that use Arabic letters without concentration on dialects, such as Aref et al. (2020), Faris et al. (2020), Omar et al. (2020), Ameur and Aliane (2021), and Khezzar et al. (2023). In other cases, researchers concentrated on certain dialects that refer to a certain region or country within the Arabian countries. This helps researchers when scraping social media, to search for keywords that are more related to this dialect. Levantine (Duwairi et al., 2021) and Gulf (Alsafari et al., 2020b; Alsafari and Sadaoui, 2021) are examples of dialects used by people in a wide region of the Arab world. So, when a researcher needs to collect data in the Levantine dialect, for example, they should add to their query the desired locations, including Jordan, Palestine, Syria, and Lebanon. If a researcher concentrates on a certain country, the location query only includes this country. Saudi (Alshaalan and Al-Khalifa, 2020; Alshalan and Al-Khalifa, 2020), Tunisian (Salomon et al., 2022), and Egyptian (Ahmed et al., 2022) are examples of such dialects. As for the Jordanian dialect, our work is considered the first to tackle data in this dialect, as far as we know.

Other query questions are heavily used by researchers when scraping social media platforms during the period. This can allow the researchers to study public opinions in a period when certain political or social events have happened.

Since scraping the social media platforms and annotating them with proper labels is not easy, it can be noticed that the size of such corpora is not considered large. Most corpora listed in Table 1 have sizes less than 10,000 annotated text, while only three exceeded this number. Thus, collecting and annotating over 400,000 tweets in our work is a vital contribution compared to other corpora proposed in the literature.

To evaluate the collected corpora, researchers have conducted hate speech detection algorithms on them. It can be noticed how most literature has concentrated on using DL algorithms, especially RNN and its variations; LSTM and GRU, in addition to DL transformer-based models, such as BERT, AraBERT, mBERT, and others. This refers to the special features of text data over other types, such as tabular ones. Extracting text features depends on the relationships and associations between words in the same text, not necessarily adjacent words. Thus, such classifiers can capture such features more efficiently than others. Nevertheless, ML classifiers have proven to efficiently use proper word embedding techniques to represent and extract the important features from a text. Table 1 summarizes the best classifiers used in the literature and their results.

### 2.3 Hate speech datasets for other languages

As aforementioned, many English hate speech corpora have been created to conduct the hate speech detection task. Recent surveys review such literature, which proposed high-quality hate speech corpora that researchers can use for further investigation (Alkomah and Ma, 2022; Yi and Zubiaga, 2023). Nevertheless, low-resource language corpora other than Arabic can be hard to find.

Table 2 illustrates information about previous research that collected corpora in low-resource languages, such as Turkish (Beyhan et al., 2022; Coban et al., 2023), Kurdish (Saeed et al., 2022), and Bangali (Romim et al., 2021).

**Table 2:** Summary of selected related literature.

**References**	**Language**	**Source**	**Dataset size**	**Labeling process**	**Classes**	**Best classifier**	**Results**
Coban et al. (2023)	Turkish	Facebook	5,000	Manual	Cyberbullying or Non-Cyberbullying (balanced)	BERT	F1-score: 92.8%
Romim et al. (2021)	Bengali	YouTube and Facebook Comments	30,000	Manual	Hate or Not hate	SVM	Acc: 87.5%
Beyhan et al. (2022)	Turkish	Twitter	Istanbul Convention Dataset (1,033), Refugee Dataset (1,278)	Manual	5-classes: no hate speech, insult, exclusion, wishing harm or threatening harm	BERTurk	F1-score: Istanbul convention dataset (71.52%), Refugee Dataset (72.34%)
Saeed et al. (2022)	Kurdish	Facebook comments	6,882	Manual	Hate or not hate	SVM	F1-score: 68.7%
Mollas et al. (2022) (ETHOS)	English	YouTube and Reddit comments	Binary (998), Multi-label (433)	Auto and Manual	8-classes: violence, directed vs generalized, gender, race, national origin, disability, sexual orientation, or religion	Binary: DistilBERT, Multi-label: NNBR (BiLSTM + Attention Layer + FF layer)	F1-score: Binary: 79.92%, Multi-label: 75.05%

Sizes, labeling process, classes, and best classifiers are displayed in the table, summarizing the important aspects of this literature.

It is worth mentioning that in high-resource languages, such as English, the researchers tend to concentrate on fine-grained classes that distinguish the types of hate speech since the number of keywords indicating these classes can be classified more easily. Consequently, this enables the researchers to create complex classifiers with multiple layers that may yield high performance (Mollas et al., 2022).

## 3 Methodology

**Figure 2:**
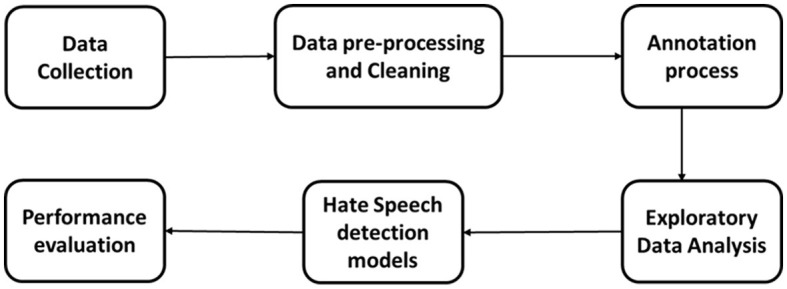
Methodology for creating JHSC and model hate speech based on it.

This section details the comprehensive methodology used to construct, annotate, and evaluate the Jordanian Hate Speech Corpus (JHSC) for detecting hate speech focused on the Jordanian dialect. Our approach includes rigorous data collection, careful pre-processing, manual annotation, exploratory data analysis, and performance assessment using hate speech detection models. The methodology used reflects the robustness, reliability, and applicability of the JSSC in developing research analyzing hate speech in the context of the Arabic language and dialects. Figure 2 illustrates the general methodology used to create JSSC and model hate speech based on it.

### 3.1 Data collection

The initial phase of constructing the Jordanian Hate Speech Corpus (JHSC) involved the collection of Arabic Jordanian dialect tweets from the Twitter platform. The tweets were collected from the beginning of 2014 to the end of 2022. To ensure the authenticity of the collected data, the following steps were applied:

1. Language Filter: The search parameters were further refined by specifying the Arabic language, ensuring that only tweets written in Arabic were retrieved.
2. Location Filter: After scrapping a random sample of tweets, it was found that most tweets do not have the location field that was supposed to be populated in users' profiles. To overcome this issue, Twitter's advanced search techniques included location-based filters. The “search” techniques focused on Jordan's main cities and regions, covering 12 governorates of Jordan and including 20 cities and regions as listed in Table 3.
3. Systematic temporal approach: The data collection process was organized over a period extending from the beginning of 2014 to the end of 2022. A monthly segmentation strategy was adopted, where tweets for each year were extracted individually and systematically monthly. This approach ensured the stability of the scrapping process and the systematic accumulation of tweets spread over a longer period. Subsequently, the distinct groups from each month and year were combined into one data set. The initial data set contained 2,034,005 tweets in the Jordanian Arabic dialect.

**Table 3:** Jordan's main cities and regions.

Abu Alanda	Ajloun	Al Karak	Al Mafraq	Al salt
Amman	Aqaba	Baqaa	Irbid	Jarash
Karak	Maan	Madaba	Mafraq	Mutah
Ramtha	Salt	Tafilah	Zarqa	Marka

It worth mentioning that the collection process targeted the public tweets only, so no privacy invasion have been conducted. As for the usage of these tweets, it should be clarified that the collected dataset is used for exploration study only, and no legal actions are entailed regarding detection of hate speech. Since, such actions should be licensed and applied by formal parties when the model is used in real life.

### 3.2 Data pre-processing and cleaning

**Figure 3:**
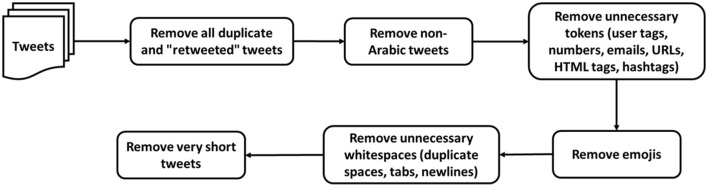
Data pre-processing and cleaning steps.

To reduce noise in the data, several steps have been performed to clean and process the dataset; data pre-processing and cleaning steps are illustrated in Figure 3. First, all duplicate and “retweeted” tweets were deleted, as recommended by Barbosa and Feng (2010) and Alayba et al. (2017). Next, the non-Arabic tweets were removed from the dataset since we focused on Arabic-Jordanian tweets. Then, unnecessary tokens such as user tags, numbers, emails, URLs, HTML tags, and hashtags were removed because they might reduce the performance of the classifier (Refaee and Rieser, 2014; Al-Twairesh, 2016).

Although emoji show feelings, they were removed from the dataset because keeping the emoticons in the dialect Arabic tweets reduces the performance of the classifier (Refaee and Rieser, 2014; Al-Twairesh, 2016), and this is due to the way Arabic sentences are written from right to left, which leads to the reversal of emoticons, as well as due to misunderstanding between brackets in the quote and emoticons. After that, all whitespaces, such as duplicate spaces, tabs, and newlines, were removed from the dataset.

Finally, the very short tweets with two or fewer words were removed from the data set. It is worth mentioning that the stemming algorithms were not applied to the dataset because they need to work better with Arabic dialect words (Al-Twairesh, 2016). After applying the pre-processing and cleaning steps, the dataset has 1,824,220 tweets.

### 3.3 Data annotation

The annotation process is pivotal in creating the Jordanian Hate Speech Corpus (JHSC). It includes careful manual tagging of each tweet with sentiment categories specifically geared toward identifying instances of hate speech. This process contributes to the development and evaluation of hate speech detection models.

#### 3.3.1 Annotation process stages

The process of Annotation tweets was done in two stages: lexicon-based annotation stage and manual annotation stage.

- Stage one—Lexicon-based annotation

In this stage, an Arabic hate lexicon from related research was used. This lexicon contains 357 terms that are considered hate or offensive terms (Mubarak et al., 2017), a sample from the lexicon term listed in Table 4. In this stage, all tweets that contain any term from this lexicon were extracted to a separate sub-dataset. The new sub-dataset contains 557,551 tweets, around 30 of the original dataset. The new sub-dataset was then processed through stage 2 of annotation.

- Stage two—Manual annotation stage

**Table 4:** Sample from bad-words lexicon.

اباد	طرد	نجس	جاهل	ابليس	حوثي
بقر	كلب	حرق	دواعش	حريم	خنزير

In this stage, the sub-dataset was labeled with four labels for sentiment: negative, neutral, positive, and very positive. The meaning and examples of each label are mentioned in Table 5.

**Table 5:** Tweets samples.

**Label meaning**	**Tweet**	**Tweet meaning**	**Label**
Tweets that have a clear indicator that the opinion is positive	یا نساء العالم كل التحیة والاحتر ام بھذا الیوم ولاكن نكن كل الاحترام والتقدیر إلى تاج نساء العالم نساء العربي	This tweet contains respect and appreciation for women all over the world and Jordanian women in specific	Negative
Tweets that are not offensive or hateful.	بضل الواحد یخطط شھر انھ كیف بده یدر س المادة	This tweet is written by a student talking about his study plans	Neutral
Tweets that are offensive but do not contain hateful content.	اطلع یا كلب یا ابن الكلب یا حامیھ یا قتلھ ھاي	This tweet contains bad words (swearing)	Positive
Tweets that contain hateful content directed at a specific group of people.	غریم الاردنیین الداعشي الكلب أبو بلال التونسي	This tweet contains bad words (swearing) that target specific names for known terrorists, and violent words such as murder	Very positive

**Figure 4:**
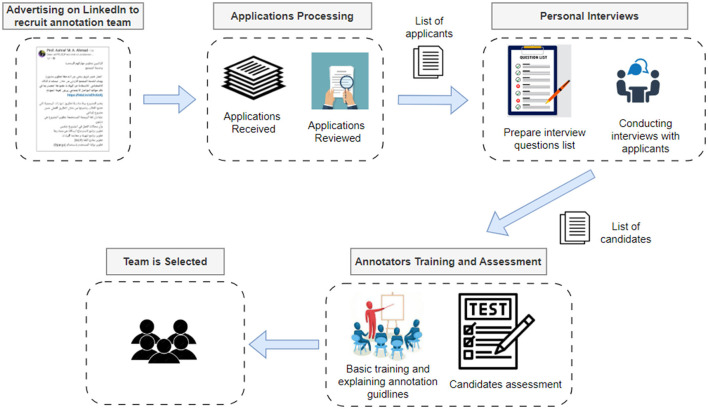
Annotation team selection.

**Figure 5:**
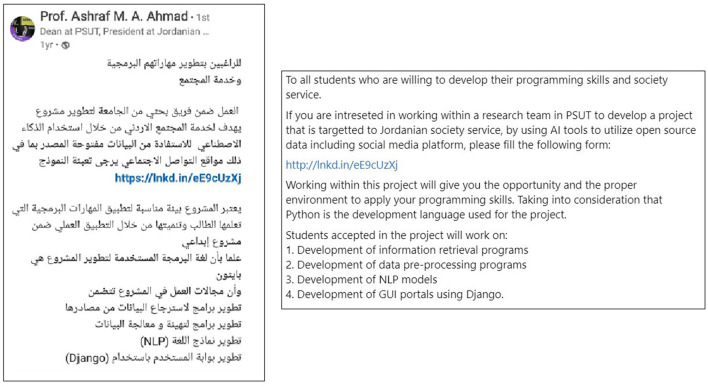
Announcement for building annotators team.

**Figure 6:**
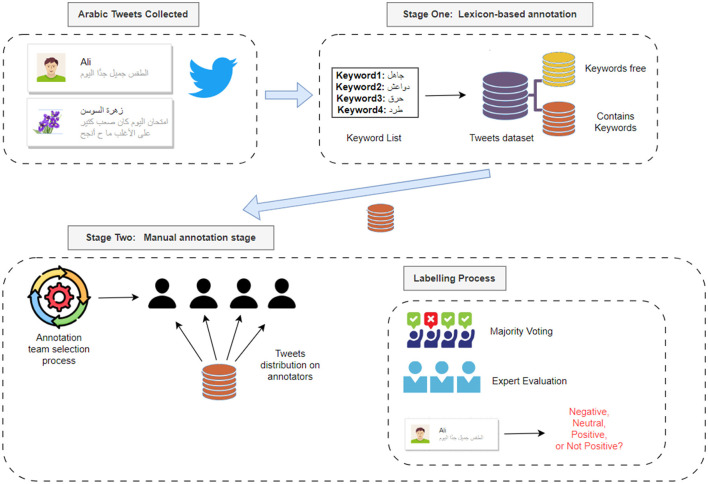
Data annotation process.

The manual annotation process is designed to ensure accuracy and agreement between annotators. This stage was performed through the following tasks:

- Task one—Annotation guidelines
  To enhance the reliability of annotations, a comprehensive annotation guideline was established, with the cooperation of domain experts, specifically in Arabic language and linguistics. This guideline outlined specific criteria and linguistic indicators for each hate speech class, guiding annotators toward consistent and accurate labeling decisions.

- Task two—Hiring annotators team
  The sub-dataset was manually annotated for hate speech by a team of annotators. Figure 4 illustrates the steps to perform this process.
  The process started with an advertisement that has been published on LinkedIn. The purpose was to find qualified personnel, mainly students, who could participate in the scraping and annotation part of the project. Figure 5 displays a screenshot of this advertisement.
  The filled applications have been reviewed, and thirty applicants have been interviewed. Twenty of them were selected after passing these interviews. A meeting has been conducted by the main researcher with the interviewed annotators to clarify the project requirements and the expectations from their side. Some candidates have been directed to work on social media scraping, while others took a quick training to understand the annotation task required. Before starting the annotation task, the candidates took a test that assessed their understanding of the annotation guidelines presented during the training and the annotation process. A link to the test is provided in Community standards enforcement report.^2^ Candidates who passed the test, scoring 70 have proceeded with the annotation process. As a start, to validate the annotation guidelines, the annotators, who were native Jordanian Arabic speakers, participated in the following phases:
  1. The annotators were given a training set of 100 tweets annotated by human experts.
  2. The annotators then independently applied the guidelines to another test set of 100 tweets.
  3. The annotators' annotations were compared with the experts' annotations. Differences were addressed through discussion. The guidelines have also been modified as necessary.
  In addition to the above, periodic meetings were held between human experts and annotators during the annotation process. Through these meetings, the work was closely followed, and issues that emerged during the annotation process were discussed and addressed, such as ambiguous expressions and discordant attitudes in the corpus, to ensure the quality of the sub-data set. The inter-annotator agreement was computed to confirm the quality. Figure 6 briefly illustrates the main steps of the data annotation process.

#### 3.3.2 Inter-annotator agreement

Inter-annotation agreement (IAA) measures how well many annotators can make the same annotation decision on the same data when doing the annotation task independently. It is an important metric in many natural language processing (NLP) tasks, such as text classification, sentiment analysis, and named entity recognition. Fleiss' kappa is an IAA statistical measure that considers the number of annotators and the number of classes. Fleiss' kappa was computed from a sample of 500 tweets annotated by three annotators to choose one of four classes: positive, neutral, negative, and very negative. The kappa rate was 0.60, indicating a moderate level of agreement between the annotators (Landis and Koch, 1977).

In summary, the use of Annotation Quality Control (AQC) through rigorous training of annotators and inter-annotator reliability checks ensures that our annotations are of high quality. This is achieved through rigorous training of annotators, as explained in the annotation team selection process, as well as the use of an Inter-annotator Agreement (IAA) to measure the extent to which multiple annotators agree on the same annotation decision when doing the annotation task independently. Additionally, the expert team provides the annotators with well-defined annotation guidelines on how to annotate the data, as explained in the Annotation process stages section, which ensures that the annotators understand the annotation guidelines and can apply them consistently. Furthermore, the majority voting and expert evaluation that deployed in the annotation process by considering the majority decision as the final annotation, and the expert evaluation provides feedback to the annotators. This helps to identify any discrepancies in the annotations and allows for corrections to be made.

### 3.4 Exploratory data analysis

**Figure 7:**
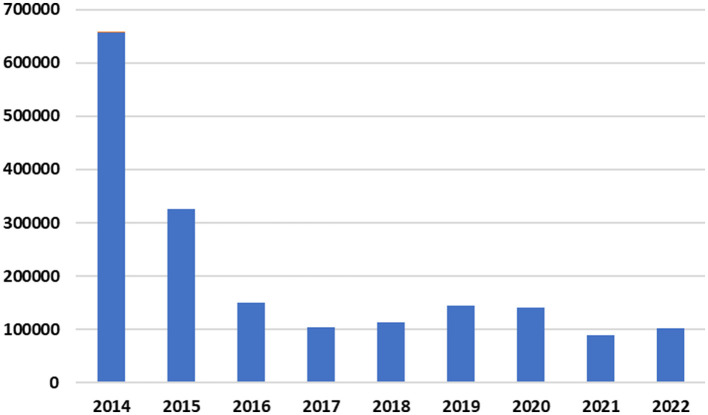
Distribution of collected tweets throughout the years 2014 to 2022.

The cleaned corpus has 1,824,220 tweets. Figure 7 shows the tweets' distribution from 2014 to 2022. It is worth mentioning that Figure 1 shows the turnout of Jordanians on Twitter in 2014, then how it decreased by half in the following year. It is also possible to note the relative stability in the past five years despite the Corona epidemic that swept the world between 2019 and 2022. In general, it is known that the epidemic increased the percentage of participation on social media platforms. Still, the reason for the decline in the participation rate of Jordanians on Twitter can be attributed to the presence of other platforms for social communication, in addition to the tightening of penalties in the cybercrime law in Jordan.

The corpus is partitioned into two parts. Part one has 1,266,669 tweets, which will be used to build the Jordanian dialect language model. Part two has 557,551 tweets used to construct the Hate speech Jordanian tweets dataset. Currently, this dataset consists of 403,688 annotated tweets, while the remainder is still undergoing the annotation process. Indeed, the dataset has 149,706 positive tweets, 126,297 offensive tweets, 7,034 very offensive tweets, and 120,651 neutral tweets.

### 3.5 Feature engineering and model construction

#### 3.5.1 Text representation

Building a Hate detection system based on machine learning and deep learning requires numerical input features. Converting words into numbers allows machines to perceive and decode linguistic patterns, which is fundamental in most NLP jobs. This process is referred to as text representation. Even if it is an iterative process, this one is crucial for selecting the features of any machine learning model or algorithm. Therefore, the input text must be first transformed into numerical features that can easily fit into machine learning algorithms.

**Figure 8:**
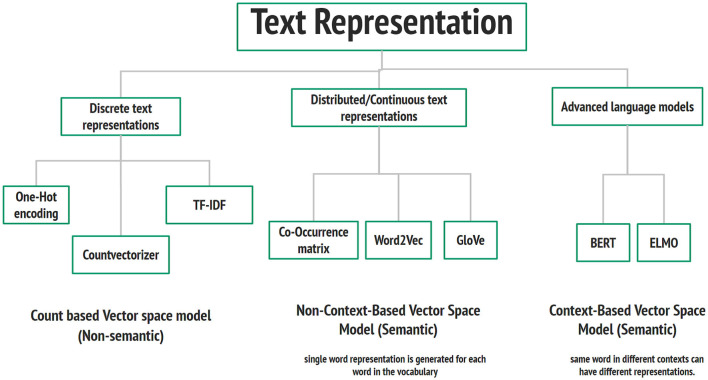
Text representation techniques.

Text representation can be divided into three sections: discrete text representation, distributed text representation, and advanced language model, as shown in Figure 8. Under each category of text representation, there are various techniques. In this paper, we focus on three popular techniques: Term Frequency-Inverse Document Frequency (TF-IDF) (Ramos et al., 2003), Word2Vec (Goldberg and Levy, 2014), and BERT text representation.

The idea behind TF-IDF is that each word's weight is determined by a word's frequency and how specific word is frequent in the whole corpus. It takes the count vectorizer (TF) and multiplies it by the IDF score. The resultant output weights for the words are low for highly frequent words like stop-words. One of the advantages of TF-IDF is it is simple and easy to understand and implement, but unfortunately, TF-IDF cannot capture the positional information of the word, and it is highly dependent on the corpus.

Word2Vec is a word embedding model that generates a vector representation of a word (Alayba et al., 2017). Each word is represented by a defined vector size that captures its semantic and syntactic relationships with other words. The architecture of word2vec consists of the input layer, one single hidden layer network, and the output layer. The network aims to learn the word embedding vector for each word by learning the embedding and context weight matrices. There are two versions of Word2Vec: Continuous Bag of Words (CBOW), which is an efficient way to use for a small dataset; the main idea behind it is to predict the middle word in the context of surrounding words. Skip-Gram, in contrast to CBOW, predicts the surrounding context words from a single word, and it is suitable for large corpus but takes more training time (Alayba et al., 2017). The most important feature of word2vec is its ability to capture the relationships between words in terms of their syntactic and semantic relationships. Still, it needs to improve and improve with out-of-vocabulary words.

Most recently, advanced text representation techniques have been proposed based on deep contextualized text representation, which allows the generated word vectors to capture the semantic meaning of the word in the text. The emergence of the Transformer and Attention model has sped up the presence of advanced text representations such as BERT and GPT models. In this paper, we used a version of the BERT model that was trained over a large corpus of Arabic Language.

#### 3.5.2 Research methodology

**Figure 9:**
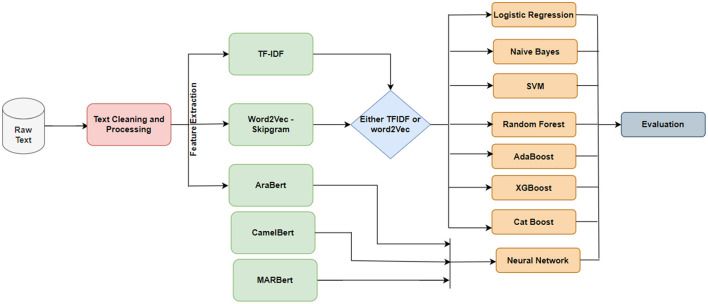
Research methodology and the experiment framework.

Figure 9 depicts the research methodology conducted in this paper. In the first part of the methodology, the collected texts have been revised and filtered by first removing retweets to avoid redundancy. Text cleaning is essential in preparing text data for NLP and machine learning models. It involves preprocessing the text to remove noise, fix structural issues, and standardize the text format; this can help improve the classification model's performance and make the text easier to work with. Text data is often messy and unstructured and can contain a variety of issues that can affect the performance of a classification model. These issues may include typos, misspellings, punctuation errors, and other irregularities that can confuse the model and make it difficult to understand the content of the text. Then, the URL addresses, emojis, and other unwanted symbols have been removed. We used a regular expression in Python to complete this job. Finally, the texts have been tokenized to prepare data for text representation.

In the second phase, the text of each message is then transformed into a numeric vector using the text representation models discussed in the previous section. We have applied TF-IDF and Word2Vec text representation techniques. In addition, we used the AraBert transformer to produce text representation. However, the latter will be only used with neural network models.

**Figure 10:**
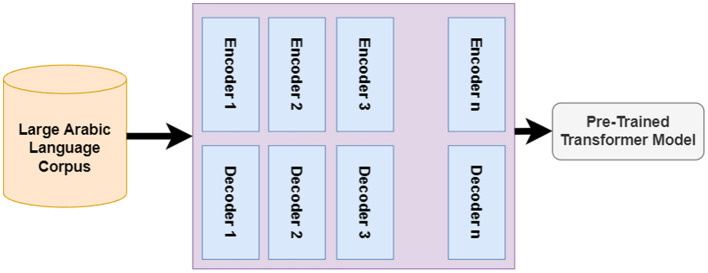
Pre-trained process of transformer.

**Figure 11:**
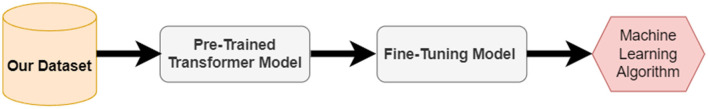
Fine tuning process.

For the AraBert model, we have used the pre-trained model as shown in Figure 10; we make fine-tuning on our corpus as shown in Figure 11. The transformer is trained over a large Arabic corpus during the pre-trained process. The output of this process is the pre-trained transformer mode, which will be used later for fine-tuning based on our collected dataset.

Numerous settings may be changed during the fine-tuning process (see Figure 11), including the optimizer, learning rate, number of epochs, and dropout value. As part of the fine-tuning procedure, we tested various optimizers, including SGD optimizer, ADAM, and AdamW. We experimented with several learning rate values, including 1 × 10^−3^, 1 × 10^−4^, and 1 × 10^−5^. We also experimented with them with several epochs ranging from 1 to 5. To prevent wasting time and storage, the terminating conditions were carefully chosen. We have tried Dropout values of 10 × 10^−2^, 25 × 10^−2^, and 50 × 10^−2^, with each number yielding a somewhat different outcome.

Finally, the text representations aligned with extracted features are entered into the NN model and placed on top of the pre-trained AraBert model. Two dense layers have been added to the NN model with ReLU activation functions. Also, a dropout layer was added to avoid overfitting during the training process, and the linear layer was used to find a correlation between input vectors and output labels. The ReLU layer will reduce the computation time required for model training. Finally, we divided the entire dataset into 70% training and 30% as testing data for validation purposes.

#### 3.5.3 Evaluation measures

Choosing proper evaluation metrics for classification problems is tricky, as every metric explains a specific part of the model performance. Wrong choices are likely to produce a poor explanation with deceived performance. Therefore, five evaluation metrics that capture different aspects of classification model predictions have been used. These metrics ensure a trade-off between the overall performance of the classification models. Since we have four class labels, we used weighted averages to aggregate all evaluation results. The most popular evaluation metrics are Recall and Precision. Recall metrics, as shown in Equation 1, can capture the proportion of hate speech correctly classified within that speech. Precision, as shown in Equation 2, is defined as the proportion of the hate speech tested as hate (see Equation 2). F1 metric, as shown in Equation 3, is used to combine the precision and recall metrics into a single metric that can work best with imbalanced data distribution. Finally, the accuracy metric shown in Equation 4 reflects the proportion of all correctly classified examples. In addition to the above metrics, we used Area Under Curve (AUC), which estimates the area under the ROC curve formed by a set of Precision and Recall values and represented as a single value in the range [0, 1]. The ROC curve presents the trade-off between recall and precision. The better model with a high AUC is regarded as the superior model.


(1) $$\begin{array}{l} \text{Recall}=\frac{tp}{tp+fn} \end{array}$$

(2) $$\begin{array}{l} \text{Precision}=\frac{tp}{tp+fp} \end{array}$$

(3) $$\begin{array}{l} \text{F1}=2\times \left(\frac{Recall\times Precision}{Recall+Precision}\right) \end{array}$$

(4) $$\begin{array}{l} \text{Accuracy}=\frac{tp+tn}{tp+tn+fp+fn} \end{array}$$

Where *tp* (true positive) is the number of hate speech predicted as such. *tn* (true negative) is the number of hate speech that is predicted as such. *fp* (false positive) is the number of not hate speech predicted as hate speech. *fn* (false negative) is the number of hate speech predicted as not hate speech.

## 4 Experiments and results

This section shows the empirical results of building hate speech detection. Three text representations models have been used to generate word vectors, namely: AraVec, TF-IDF, and AraBert model. AraVec is a Word2Vec model trained over large Arabic corpus. AraBert is Bert alike transformer which has been trained over large Arabic corpus. These models can give us the text representation as vectors to feed into classification models. We have used seven machine learning classifiers: Support Vector Machine (SVM), Logistic regression (LR), Naive Bays (NB), Random Forest (RF), AdaBoost (Ada), XGBoost (XGB), and CatBoost (CatB).

### 4.1 Experimental setup and hyperparameter tuning

Table 6 shows each classifier's searching parameters and best parameters. We identified a list of values for each configuration parameter, then we used the Grid search algorithm with 5-fold cross validation to select best configurations for each classifier.

**Table 6:** Results of negative label.

	**Recall**		**Precision**		**F1**	
	**W2V**	**TF-IDF**	**W2V**	**TF-IDF**	**W2V**	**TF-IDF**
LR	0.60	0.64	0.55	0.45	0.57	0.53
NB	0.39	0.30	0.58	0.53	0.47	0.38
RF	0.59	0.53	0.56	0.50	0.57	0.51
SVM	0.61	0.65	0.54	0.45	0.57	0.53
AdaBoost	0.56	0.59	0.54	0.46	0.55	0.52
XGBoost	0.58	0.54	0.57	0.50	0.58	0.52
CatBoost	0.59	0.55	0.57	0.51	0.58	0.53

### 4.2 Results

To investigate the quality of the collected data in addition to the quality of the annotation process, we conducted comprehensive experimentation on building a hate detection system using multiple machine learning algorithms and two main text representation techniques: Word2Vec (W2V) and TF-IDF in addition, we used BERT based Arabic language called AraBert. As explained in the research methodology, we split the dataset into 70% training and 30% testing; then, we used seven machine learning algorithms and training datasets to build different classifiers on features extracted from W2V and TF-IDF techniques. Finally, all constructed models have been evaluated on testing using multiple evaluation metrics.

To facilitate presenting the results, we organized all results into different tables based on the class labels in the dataset. Since we have four classes, we showed the evaluation results for each class label. Each table shows each machine learning performance with each text representation technique. Then, we added a table to summarize the overall results using the weight average aggregation method. Table 6 shows the evaluation results for the Negative class label. We omitted accuracy and AUC metrics because they are aggregated and not calculated individually for each class label. The bold text represents the best results between TF-IDF and W2V for each evaluation metric. The bold and red text represents each evaluation metric's best machine-learning model. From the table, we can generally observe that W2V is more suitable for our text than TF-IDF. It is widely acknowledged that W2V can produce good text representation when the corpus contains over 25,000 vocabularies, as in our case. Therefore, the machine learning algorithms that use W2V produce better results than TF-IDF ones. On the other hand, if we look at the machine learning algorithm, we notice instability in terms of performance, such that we cannot identify the best model. However, for the Recall metric we can see that SVM+TF-IDF is the best, whereas for precision, we can see that NB+W2V is the best. This contradiction forces us to choose multiple options as good candidates. Finally, the best recall accuracy for (SVM+TF-IDF) suggests that the model can predict 65%.

Table 7 presents results for the Neutral class label. Generally, the results are poor because the best recall or precision score is relatively low. Interestingly, we can observe a stable result here, more than the Negative class label. Also, we found that W2V always produces good text representation for all machine learning models. If we look at the evaluation results between W2V and TF-IDF, we can see a big difference, suggesting that TF-IDF is inappropriate for such kind of hate speech corpus. Concerning the machine learning model, we cannot identify one best mode but multiple ones according to the evaluation metrics. For example, NB+W2V can work well under the Recall metric, whereas CatBoost+W2V can work well under the Precision metric. If we take the F1 metric as a compromised solution, we can see that NB and CatBoost with W2V are the best models.

**Table 7:** Results of neutral label.

	**Recall**		**Precision**		**F1**	
	**W2V**	**TF-IDF**	**W2V**	**TF-IDF**	**W2V**	**TF-IDF**
LR	0.35	0.21	0.42	0.37	0.38	0.27
NB	0.47	0.27	0.37	0.36	0.41	0.30
RF	0.39	0.36	0.42	0.37	0.40	0.36
SVM	0.34	0.21	0.43	0.37	0.38	0.26
AdaBoost	0.31	0.23	0.40	0.35	0.35	0.28
XGBoost	0.38	0.34	0.43	0.37	0.40	0.35
CatBoost	0.38	0.34	0.44	0.38	0.41	0.36

Table 8 presents results for the Positive class label. We see the same trend as the Neutral class label but with different best machine learning models. First, we can confirm that the W2V is a good text representation among all models, and CatBoost is the most accurate and stable model under three evaluation metrics. The positive Label's overall results are good compared to the Neutral label and show good performance.

**Table 8:** Results of positive label.

	**Recall**		**Precision**		**F1**	
	**W2V**	**TF-IDF**	**W2V**	**TF-IDF**	**W2V**	**TF-IDF**
LR	0.53	0.37	0.47	0.39	0.50	0.38
NB	0.47	0.27	0.42	0.36	0.45	0.30
RF	0.52	0.36	0.48	0.37	0.50	0.36
SVM	0.52	0.36	0.48	0.38	0.50	0.37
AdaBoost	0.52	0.41	0.44	0.38	0.48	0.40
XGBoost	0.56	0.43	0.49	0.41	0.52	0.42
CatBoost	0.57	0.44	0.49	0.42	0.53	0.43

**Figure 12:**
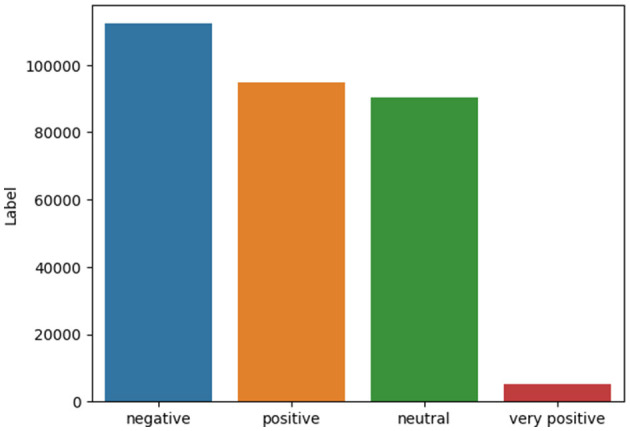
Class distribution of class label.

Finally, the evaluation results for the “Very Positive” class label are very poor, as shown in Table 9. One reason for that is the relatively imbalanced dataset's nature, which means that there is a big difference in the number of samples in each class label. Figure 12 shows the class distribution of our dataset. We can notice there is an imbalanced distribution between class labels. The “very Positive” class label is the minor one. Therefore, the performance of machine learning over this label was very poor, as shown in Table 9. Also, there are no stable results across all evaluation metrics. Therefore, judging which machine learning model is superior isn't easy.

**Table 9:** Results of very positive label.

	**Recall**		**Precision**		**F1**	
	**W2V**	**TF-IDF**	**W2V**	**TF-IDF**	**W2V**	**TF-IDF**
LR	0.00	0.00	0.00	0.00	0.00	0.00
NB	0.08	0.01	0.07	0.02	0.07	0.01
RF	0.02	0.04	0.37	0.34	0.04	0.07
SVM	0.00	0.00	0.26	0.00	0.01	0.00
AdaBoost	0.00	0.00	0.06	0.00	0.00	0.00
XGBoost	0.03	0.02	0.44	0.52	0.06	0.04
CatBoost	0.03	0.01	0.44	0.65	0.06	0.01

We aggregate all evaluation results using a weighted average that considers the class distribution with the final calculation shown in Table 10 to get insights from the above results. We can see that W2V is generally the best text representation for our corpus. All machine learning models behave relatively accurately with good performance. Amongst them, CatBoost is the most stable and accurate model.

**Table 10:** Aggregated results for all labels using weighted average.

	**Recall**		**Precision**		**F1**		**Accuracy**		**AUC**	
	**W2V**	**TF-IDF**	**W2V**	**TF-IDF**	**W2V**	**TF-IDF**	**W2V**	**TF-IDF**	**W2V**	**TF-IDF**
LR	0.49	0.42	0.48	0.40	0.48	0.39	0.49	0.42	0.48	0.50
NB	0.43	0.38	0.46	0.41	0.44	0.37	0.43	0.38	0.48	0.47
RF	0.50	0.43	0.49	0.43	0.49	0.43	0.50	0.43	0.49	0.49
SVM	0.49	0.41	0.48	0.39	0.48	0.41	0.49	0.41	0.49	0.50
AdaBoost	0.47	0.40	0.46	0.41	0.46	0.40	0.47	0.41	0.41	0.46
XGBoost	0.50	0.43	0.50	0.44	0.50	0.43	0.50	0.44	0.48	0.50
CatBoost	0.51	0.44	0.51	0.44	0.50	0.44	0.51	0.44	0.47	0.50

Concerning the Transformer Models, we finetuned three Arabic language transformers (AraBERT, MARBERT, and CamelBERT) on our Arabic hate speech corpus; then, we built a neural network model based on the CLS embedding. It is important to note that the transformer usually uses its tokenizer, producing its text representation as output through CLS embedding. Then, this embedding vector is connected to the Neural network fully. The model has been evaluated over testing corpus using the same evaluation measures shown in Table 11. We can see the transformers, especially CamelBERT, can learn text representation better than W2V and TF-IDF techniques and produce good results compared to the weighted average results of the machine learning models.

**Table 11:** Results of fine-tuned transformer model.

**Fine-tuned model**	**Label**	**Recall**	**Precision**	**F1**	**Accuracy**	**AUC**
AraBERT	Negative	0.64	0.61	0.63	0.62	0.66
	Neutral	0.43	0.55	0.49	0.62	0.63
	Positive	0.62	0.61	0.61	0.62	0.62
	Very positive	0.16	0.46	0.26	0.62	0.55
	Aggregate	0.6	0.6	0.6	0.62	0.63
CamelBERT	Negative	0.68	0.64	0.66	0.61	0.69
	Neutral	0.47	0.56	0.51	0.61	0.67
	Positive	0.67	0.6	0.64	0.61	0.68
	Very positive	0.19	0.44	0.27	0.61	0.55
	Aggregate	0.61	0.6	0.6	0.61	0.63
MARBERT	Negative	0.65	0.63	0.64	0.6	0.67
	Neutral	0.44	0.57	0.5	0.6	0.65
	Positive	0.63	0.63	0.62	0.6	0.65
	Very positive	0.17	0.48	0.27	0.6	0.53
	Aggregate	0.61	0.62	0.61	0.6	0.62

To conclude, the collected data and annotation process was very appropriate, and the obtained evaluation results show good performance for this complex and unstructured domain. We also should pay attention to the complexity of processing Arabic text, especially in Processing the natural Arabic language. For example, the word spelling can differ from one sentence to another, which changes the meaning, and there are many different Arabic dialects, even in the same country, which makes it harder to understand the meaning of the sentence; the word diary can also change the meaning.

## 5 Conclusion and future work

In this study, we address the intricate challenge of Hate Speech Detection in Arabic, a language with a wide variety and nuanced cultural characteristics. This study intends to aid in the fight against hate speech in Arabic that is spread online. A notable resource in this field is the creation of a fresh multi-class Arabic dataset with over 400,000 annotated tweets that have been sentimentally classified. Additionally, using text representation techniques, including WordVec, TF-IDF, and AraBert, and seven machine learning classifiers, we evaluated the effectiveness of several machine learning models in detecting hate speech in tweets written in the Arabic Jordanian dialect. Our empirical findings indicated our dataset's usefulness and precisely how hate speech could be identified in this difficult, unstructured environment. Although this work makes significant advancements in the Arabic Hate Speech Detection field, several areas still might be used for more investigation.

In the future, we want to increase the size and diversity of our dataset, by including Arabic dialects in addition to Jordan and neighboring countries dialect, improve contextual analysis, create real-time detection systems, look into user-specific detection, and address bias and fairness concerns. By promoting a safer online environment, these initiatives will help develop more effective and culturally relevant solutions for addressing hate speech in Arabic.

Another suggested work line in the future is to examine multilingual and cross-lingual models. Nevertheless, several challenges should be considered when tackling such problems. Some of these challenges are: the performance variability between high and low-resourced languages, the possibility of loosing language-specific nuances, in Arabic for example, when using pre-trained models on other different languages. In addition to some generalization challenges between languages that differ in structures, writing styles and other characteristics.

## Data availability statement

The datasets presented in this study can be found in online repositories: “Arabic Hate Speech Dataset 2023”, Mendeley Data, V2, doi: 10.17632/mcnzzpgrdj.2.

## Author contributions

AAh: Conceptualization, Funding acquisition, Methodology, Writing—original draft, Writing—review & editing. MA: Conceptualization, Methodology, Software, Writing—original draft, Writing—review & editing. EA: Conceptualization, Software, Writing—original draft, Writing—review & editing. QA: Conceptualization, Methodology, Resources, Writing—original draft, Writing—review & editing. DH: Formal analysis, Validation, Writing—original draft, Writing—review & editing. AAr: Writing—original draft, Writing—review & editing. YA: Visualization, Writing—original draft, Writing—review & editing.

## Footnotes

^1^Annotation exam. Available online at: https://forms.gle/9e56l2j8vh9mnsiv9.

^2^Community standards enforcement report. Available online at: https://transparency.fb.com/reports/community-standards-enforcement/hatespeech/facebook/ (accessed May 9, 2023).
